# Aging and atrial fibrillation: a matter of fibrosis

**DOI:** 10.18632/aging.102501

**Published:** 2019-11-21

**Authors:** Susana Ravassa, Gabriel Ballesteros, Javier Díez

**Affiliations:** 1 Program of Cardiovascular Diseases, CIMA, Universidad de Navarra, Pamplona, Spain; 2 IdiSNA, Navarra Institute for Health Research, Pamplona, Spain; 3 CIBERCV, Carlos III Institute of Health, Madrid, Spain; 4 Department of Cardiology and Cardiac Surgery, Clínica Universidad de Navarra, Pamplona, Spain; 5 Department of Nephrology, Clínica Universidad de Navarra, Pamplona, Spain

**Keywords:** atrial fibrillation, biomarkers, collagen, fibrosis

Atrial fibrillation (AF), the most common cardiac arrhythmia, is associated with high morbidity and mortality. It is well known that both the prevalence and incidence of AF increase sharply with age, particularly after 65 years of age. AF and aging share mutual bidirectional relationships. On the one hand, aging and aging-related underlying diseases result in myocardial remodeling that may lead to cardiac electrical abnormalities which enhance the occurrence or persistence of AF [1]. On the other hand, AF worsens biological aging, specifically at the brain level, causing injuries related to ischemic and non-ischemic events, thereby impairing functional capacity. In addition, handling of AF is challenging in aged patients due to the high prevalence of complex clinical features (i.e. heart failure [HF] and chronic kidney disease) and the progressive AF-mediated aggravation of degenerative processes typical of aging. All these aspects have profound effects on the patient health condition and on the resources provided by the society and national health systems to dedicate to the care of elderly patients.

Even though it is well known that age is the single most important determinant of AF risk, the underlying mechanisms are not completely understood. Some of the mechanisms involved in the aging-AF association may be related with age-dependent left atrial dilation or senile amyloidosis that alter the structure of the myocardial tissue and constitute typical features of the so-called AF substrate [2]. In addition, resting membrane potential depolarization and spontaneous calcium releases from the sarcoplasmic reticulum, among others, might promote afterdepolarization and trigger AF [3]. Since fibrosis is a prominent lesion present in the atria of AF patients and atrial fibrosis can both affect the substrate and induce the trigger, this lesion emerges as a factor that may play a central role in aging-related AF. In particular, by increasing the severity of atrial fibrosis, age may contribute to the development of electrical conduction disturbances and ectopic activity, affecting atrial arrthythmogenity [1].

Myocardial fibrosis is characterized not only by an excessive accumulation of collagen fibers (namely, type I fibers) in the interstitial space but also by an increased insolubility and stiffness of the deposited fibers due to an exaggerated degree of intermolecular covalent linkage, or cross-linking within these fibers. It has been reported that HF patients carrying simultaneously these two histomolecular alterations exhibit more severe cardiac dysfunction and worst clinical evolution than patients in whom the two myocardial alterations are not coincident [4]. Therefore, this particular phenotype of MF has been considered as a malignant phenotype of myocardial fibrosis (mMF).

**Figure 1:**
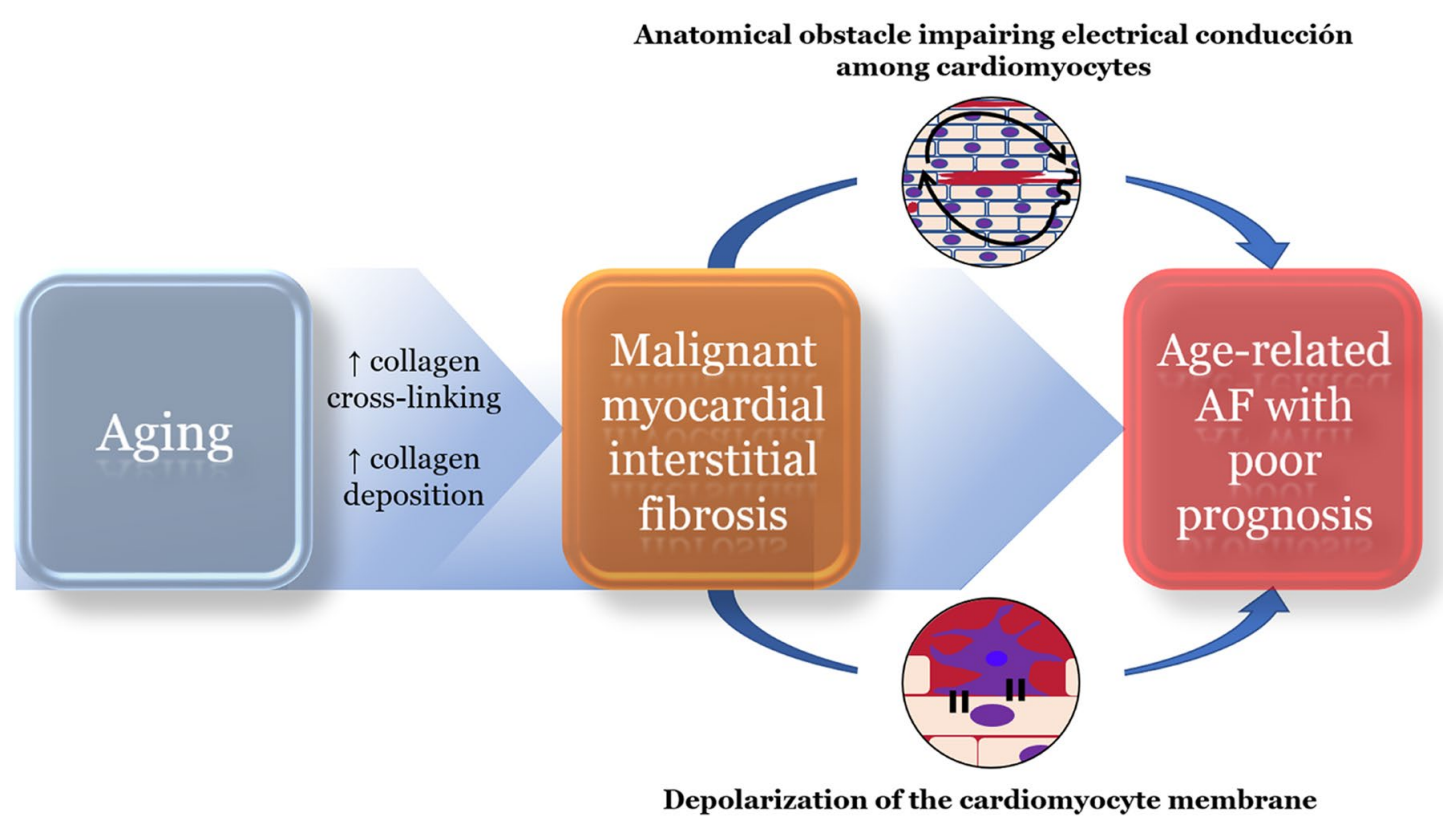
**Proposed role of malignant myocardial fibrosis in aging-related atrial fibrillation (AF)**. An excessive myocardial collagen type-I deposition and cross-linking can form an anatomical obstacle of dense fibrosis (red color in the upper circle) and induce slow and heterogeneous conduction around it (black arrow in the upper circle), typical alterations of the arrhythmogenic substrate. Also, due to myofibroblast-cardiomyocyte coupling (lower circle) this particular form of fibrosis can partially depolarize the cardiomyocyte cell membrane, inducing both triggered activity and abnormal automaticity. In this way, malignant myocardial fibrosis fulfills the two key elements of the pathophysiology of AF, a triggering arrhythmia for its initiation and an impaired atrial conduction to perpetuate its perpetuation.

The presence of mMF can be biochemically assessed by using a combination of circulating biomarkers related to collagen type I metabolism. On the one hand, the ratio of serum carboxy-terminal telopeptide of collagen type I (CITP) to serum matrix metalloproteinase-1 (MMP-1) (CITP:MMP-1 ratio) has been shown to be inversely correlated with left ventricular myocardial collagen type I cross-linking in HF patients, as the higher is the cross-linking among collagen type I fibrils, the lower will be the cleavage of CITP by MMP-1 during the process of degradation of the fiber [5]. On the other hand, the serum carboxy-terminal propeptide of procollagen type I (PICP), released during the conversion of procollagen type I into fibril-forming mature collagen type I, has been reported to be directly correlated with left ventricular myocardial collagen type I deposition in HF patients [6]. Interestingly, the combination of low CITP:MMP-1 ratio and high PICP reflecting the presence of mMF has been associated with higher AF prevalence, incidence of AF and with AF recurrence after ablation procedure in patients with HF [7]. In addition, patients with AF presenting with the combination of biomarkers of mMF have been shown to exhibit lower atrial voltage as compared with patients without the combination [7]. Of interest, a sub-analysis of the previously mentioned data [7] comparing AF patients <50 years old and AF patients >75 years-old reveals that compared to the former the later exhibit a higher frequency of the combination of biomarkers of mMF (50% vs 13.3%, P=0.045), a more frequent recurrence of AF (60% vs 20%, P=0.041), a lower mean atrial voltage (median [interquartile range]: 0.38 [0.27-0.74] mV vs 1.26 [0.86-2.24] mV, P=0.008) and higher values of the slope of the voltage histogram, a recently described parameter potentially related to atrial electrical or structural heterogeneity [8] (0.87 [0.72-2.38] vs 0.58 [0.40-0.65], P=0.014). These data suggest that aging may perpetuate AF and influence the response to the ablation treatment in patients with this pathology by, among other mechanisms, promoting the presence of mMF and subsequent worsened electrical disturbances in the atria (Figure 1).

Further studies are required to ascertain whether the use of biomarkers of mMF, as a non-invasive tool to detect the fibrotic-related structural abnormalities that affect atrial electrical properties, may help to stratify the risk of adverse clinical evolution in elderly patients with AF. In addition, it would be necessary to establish whether specific therapeutic strategies to correct myocardial collagen metabolism in aged AF patients with the mMF biomarker profile may help with their clinical handling.
